# Association of Place of Residence and Under-Five Mortality in Middle- and Low-Income Countries: A Meta-Analysis

**DOI:** 10.3390/children5040051

**Published:** 2018-04-18

**Authors:** Ian Forde, Vrijesh Tripathi

**Affiliations:** 1 Foundation and Prior Learning, University of Trinidad and Tobago, Wrightson Rd, Port of Spain, Trinidad and Tobago; ianforde.snr@gmail.com; 2 Department of Mathematics and Statistics, The University of the West Indies, St. Augustine, Trinidad and Tobago

**Keywords:** rural, urban, under-five mortality (U5M), developing countries, meta-analysis

## Abstract

This review evaluated the association of place of residence (urban/rural) and under-five mortality in middle- and low-income countries. Both English and Spanish language studies conducted during the Millennium Development Goal (MDG) period (1990 to 2015) were reviewed. Twenty-six cross-sectional studies, all in the English language, were selected for further review. Published data were used for this analysis. A funnel plot was produced to ascertain the presence of publication bias. The combined relative risk for under-five mortality was estimated using a random-effects model and a meta-regression was conducted on 15 of the 26 studies. The studies had a combined effect size of 1.47 (95% confidence interval, 1.27–1.67). The results of the meta-regression showed a positive association between the relative risk and the percentage of the rural population for the various regions/countries. The coefficient for the variable rural population percentage was 0.007, indicating that for every one percent increase in the rural population percentage, there was a 0.007 increase in the relative risk for under-five mortality. However, this was not significant (*p*-value = 0.3). Rural disadvantage persists in middle- and low-income countries. This is important to evaluate policies and programmes designed to remove the gap in under-five mortality rates between urban and rural areas.

## 1. Introduction

Under-five mortality (U5M) is defined as a child dying between birth and their fifth birthday. In 2015, 16,000 children under the age of five died every day, approximating 5.9 million child deaths for the year [1]. The global U5M at the beginning of the Millennium Development Goals (MDGs) era was 91 per 1000 live births in 1990. This reduced by 53% to 43 per 1000 live births in 2015 [2]. Despite this decrease, many countries did not achieve MDG4, which had set the target of reducing U5M by two-thirds between 1990 and 2015. The United Nations has set newer Sustainable Development Goals (SDGs) to be achieved by 2030. Of particular interest to the present study, are SDGs 3 and 10, which aim to reduce inequalities within and among countries, ensure healthy lives and promote well-being for all ages [3]. There are a number of known maternal factors, including maternal wealth quintile, age and education that are known to be associated with U5M [4,5]. Rural/urban place of residence is an established predictor of U5M. In traditional societies in the nineteenth and early twentieth centuries, rural populations had a distinct advantage over urban populations in U5M, but the advantage was reversed in decolonized countries in the latter half of the twentieth century. Overall, since the 1950s, urban areas are associated with lower U5M than rural areas [6,7]. This seems counter-intuitive since rural areas have less over-crowding, are spacious, have less air pollution, less spread of epidemics and have access to clean water and sanitation facilities. However, the health care sector is more developed in urban than in rural areas. Also, there is higher a prevalence of mother’s educational level, awareness level, socio-economic status and lower social taboos in urban than in rural areas. This presents a confusing picture, as it has also been reported that overcrowding in urban areas brought about by rapid urbanization has led to the reversal of the advantage [8]. Distance from the nearest health facility causes low use of institutional health facilities in rural areas. Hence, we have undertaken this review to determine the association between rural place of residence and U5M.

A systematic review of the available literature at this juncture provides three main benefit: first, to evaluate the evidence for an association between rural place of residence and U5M; second, to assist in how best to allocate limited resources for combating U5M in middle- and low-income countries; and third, to assess the effectiveness of the strategies adopted prior and during the MDG era in removing the disadvantage in rural areas. The MDG era is an excellent point in time to use to conduct this review because it was a period when many of the middle- and low-income countries engaged in efforts to reduce their respective U5M. The present review, therefore, determined whether the efforts employed removed the rural/urban gap in U5M in middle- and low-income countries.

## 2. Materials and Methods

The question that guided the review was “Is there an association between place of residence (rural/urban) and U5M in middle- and low-income countries?” The review included articles published in electronic databases, including Pubmed and WHOLIS. Additional sources of articles not published by journals but posted online were also accessed through Google Scholar. Both English and Spanish language articles were accessed and the translation of the Spanish article was done with the assistance of Google Translate. The studies were then referred to speakers of the Spanish language for a second opinion. A search was conducted online by the first author, initially, using the keywords or phrases “rural-urban”, “difference” and “under-five mortality”. To narrow the search results, the phrase “developing countries” was then added. For example, the phrase “rural and urban differences in under five mortality in developing countries” was searched in Pubmed. This returned seven items from which one study, “Socioeconomic and geographical disparity in under-five mortality and neonatal mortality in Uttar Pradesh, India”, was selected for a review of its abstract [9]. Variation of the words or phrases were also used in the subsequent searches, for instance “rural–urban gap” was used in a third search in an attempt to identify studies that might have been missed in the first two searches.

**Figure 1:**
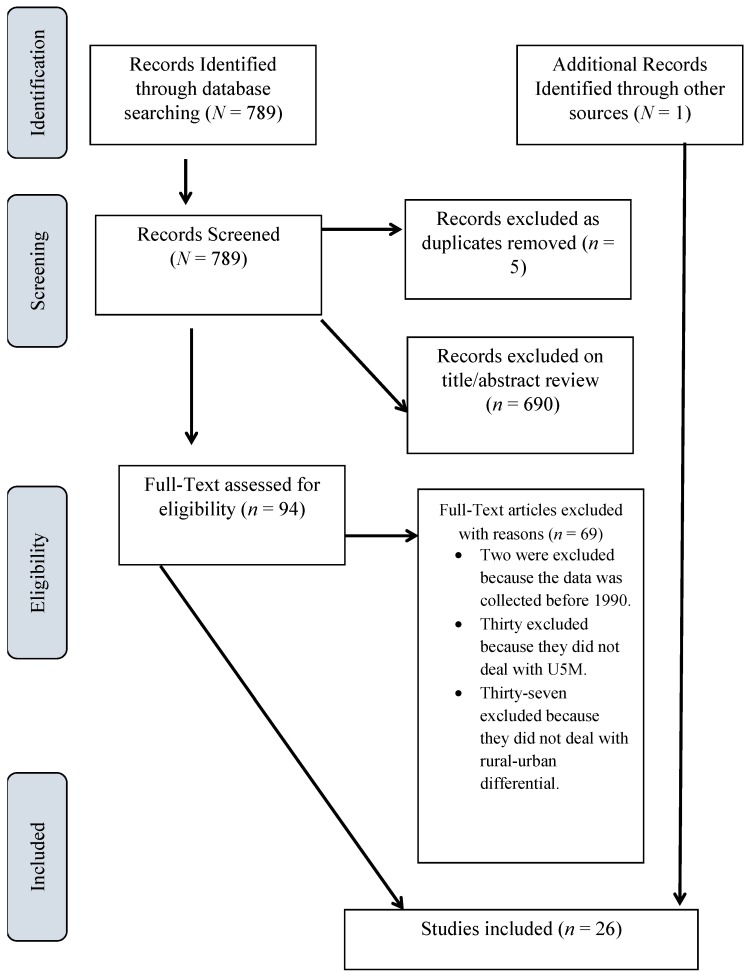
Flow diagram of study selection including reasons for exclusion for under-five mortality (U5M).

An initial title search yielded 789 studies from the various databases and one from an additional source, the University of the West Indies, St. Augustine library (St. Augustine, Trinidad W.I.). The titles of the articles selected indicated that the rural-urban differential in U5M was the main concern of the studies. The results of the search are presented below (Figure 1), together with reasons for exclusion of studies. Following the initial title search, a review of abstracts was done and this returned 94 studies which were selected for full text review. All the studies selected were in the English language, conducted in the MDG period from 1990 to 2015. This is in keeping with Hartling et al.’s study that suggested studies of non-English and unpublished sources represent a small proportion of included studies and rarely impact the results and conclusions of systematic reviews [10]. Studies that did not provide comparative understanding into the rural-urban U5M were excluded. Studies that dealt solely with infant mortality or solely with child mortality or with infant and child mortality without combining the two were excluded. All studies included used place of residence as a categorical variable (urban/rural). Twenty-six studies met the stipulated eligibility criteria. The selected studies were mainly global studies in middle- and low-income/developing countries in Sub-Saharan Africa, South and Southeast Asia, and Latin America/Caribbean. One study was conducted in the Southwestern Pacific region (Table 1). All the selected studies used cross-sectional data from the demographic health surveys (DHS), census or birth registries of the respective countries/regions. This ensured that no study was at a disadvantage in terms of the source of bias due to study design.

Published data were extracted from the full text articles of the 26 studies using a data extraction form. The form consisted of fields that required data such as reference (First author/Year/Journal citation), location, period of study, population focus, study design, main outcome measure, results, quality score and notes. Given the small number of studies selected, no coding was necessary. The data were entered from the data extraction form directly into Microsoft Excel 2010 (Microsoft Corporation, Redmond, WA, USA) for further analysis.

The quality of the individual studies was assessed on their study design, the quality of conduct and the quality of reporting. The first author did an assessment with input from the second author. The scale used for the quality of conduct and reporting was good, fair or poor. In addition, the studies were also assessed for adjustment for confounding variables. If a study looked at the trend of the U5M by place of residence in the presence of other confounding variables such as wealth quintile, the study received a “yes” rating. An overall summary judgment was also done using a scale similar to that used to assess conduct and reporting. All of the selected studies obtained an overall good rating. A good rating meant that the design and conduct of the study minimized risk of bias and outcomes were adequately measured using appropriate analytical methods and tools.

Meta-analysis was done on 15 of the 26 studies. Published results from each study were combined. The studies included in the meta-analysis met the following criteria: they were original epidemiological studies whose populations were clearly defined; U5M was defined as a child dying between birth and the fifth birthday; all the studies reported counts, rates or relative risks (RR) for U5M for both rural and urban areas; the independent variable was place of residence (rural/urban) of the mother at the time of the survey; and the response variable was U5M, from which the various comparative measures of U5M, including the RR and absolute difference, were calculated. Where the relative risk was not reported it was determined by entering the U5M rate in an online statistical calculator that utilizes the method described by Altman in 1991 [11]. Summary tables were double-checked to confirm data accuracy.

A step-by-step guide constructed by Neyeloff et al. to conduct a meta-analysis in Excel 2010 (Microsoft Corporation) [12] was used as a guide to conduct this meta-analysis. The 11 studies not included in the meta-analysis reported measures that were not common to the other studies. The analyses involved combining the results of the selected studies in a random effects model to obtain an effect size that was representative of all the studies. A random effects model was best suited for the present review because it assumes between-study variations, such as differences in study sample.

Effect sizes of individual studies were directly extracted from the publications and used to calculate the combined effect size in the meta-analysis. No pooling of data was done. The *I^2^* index was used to assess consistency between studies, as it does not inherently depend on the number of studies in the meta-analysis. As suggested by Higgins et al. the *I*^2^ index was interpreted as low inconsistency if equal to 25% or less [13]. To address the possibility of publication bias, we examined funnel plots. Sensitivity analysis was done by omitting each study in turn and rerunning the meta-analysis each time to determine the effect on the combined relative risk.

A random effects meta-regression analysis was also carried out on 15 studies [14]. The response variable was the relative risk of U5M by place of residence and the independent variable was the percentage of rural population of the various territories under study. The percentage of rural population was determined for the year the data was collected from the World Bank Data page [15]. Where the territory consisted of more than one country, the average of the rural population percentage was used. A normal probability plot was used to test the assumption that the effect outcomes were normally distributed. All calculations were done with Excel 2010 (Microsoft Corporation).

### Ethical Considerations

This systematic review used published data from studies. Since no raw data were analyzed, there was no requirement for ethical approval.

## 3. Results

**Figure 2:**
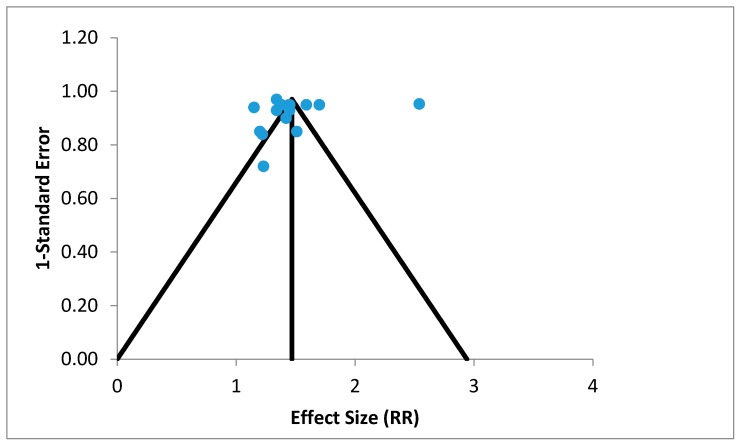
Funnel plot of effect sizes of the 15 selected studies (random effects model), RR: relative risk.

Overall, 26 studies were eligible for this systematic review; these studies are described in Table 2. Nearly half the studies were done at country, province and/or district level, while the rest were multi-country studies. Of all the 26 studies selected, 23 studies reported a significant rural disadvantage (*p*-value < 0.05), indicating that U5M was higher in rural areas. The effect sizes of the 15 studies were plotted on a funnel plot to analyze asymmetry (Figure 2). Asymmetry may be due to significant publication bias, between study heterogeneity and chance. Reporting bias and chance are suspected since there was a lack of symmetry in the funnel plot (Figure 2). Since studies appeared to be missing in the lower part of the plot, publication bias is plausible. Indeed, it may more likely be due to reporting bias; since heterogeneity was low, *I*^2^ was determined to be 0% despite the fact that a number of the studies included sub-studies.

**Figure 3:**
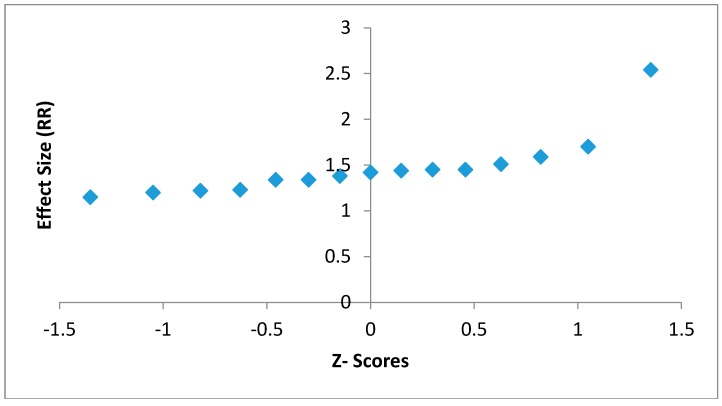
Normal probability plot to test for normality of the effect outcomes of the 15 studies.

The assumption of normality held true since there were not many deviations from a straight line observed in the normal probability plot (Figure 3).

**Figure 4:**
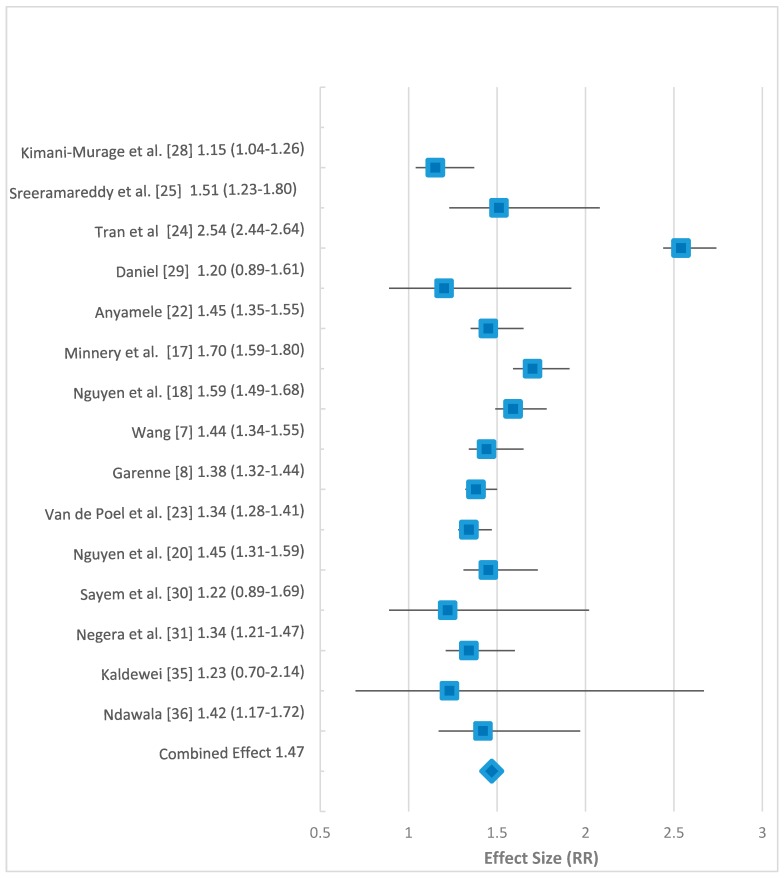
Forrest plot of effect sizes, (relative risk (RR) with 95% confidence interval) for the 15 studies used in the meta-analysis.

The effect sizes for the 15 studies used in the meta-analysis ranged from 1.15 in the Kimani-Murage study to 2.54 in the Tran study [24,28]. Twelve of the studies had significant results (*p*-value < 0.05). Three studies done by Daniel, Sayem and Kaldewei had RR greater than 1 (1.2, 1.22 and 1.23, respectively) but did not find the place of residence significant at *p*-value < 0.05 [29,30,35]. When the results from all the studies were combined, the RR of U5M was higher for those living in rural areas. The overall combined effect size was determined to be 1.47 (95% confidence interval (CI): 1.27–1.67). Results of the 15 studies together with the combined effect size are displayed in the following Forrest plot of the association between rural place of residence and U5M (Figure 4).

The meta-regression showed that there was a positive relationship between the percentage of rural population for the various countries/regions and the relative risk for U5M by place of residence. The beta coefficient (*β*) for the rural population percentage was 0.007. This means that for every one percent increase in the rural population, there was a 0.007 increase in the U5M. This, however, was not significant (*p*-value = 0.3). The 95% confidence interval for the coefficient was (−0.006, 0.02) (Table 3).

Sensitivity analysis, where individual studies were omitted, demonstrated that rural disadvantage was evident each time. Relative risk reduced when the Minnery et al. [17] and Tran [24] studies were omitted (Table 4). In all instances, heterogeneity remained at 0%.

## 4. Discussion

The overall results showed fairly consistently that the relative risk of U5M increases for those living in rural areas. When the results from 15 studies were combined, the findings were broadly similar for U5M being higher for rural areas than urban areas. It is evident from our review that rural disadvantage persists in U5M. The fact that researchers have overwhelmingly reported that rural place of residence is associated with increased U5M means that the policies and programmes implemented to remove rural disadvantage must be assessed. Many studies indicated that inequalities decreased between urban and rural place of residence [20,25,27,28,38], but some also indicated a widening of the gap [24,26]. While some studies reported that the decline in U5M was faster in urban areas [7,24,30], others reported there was lower progress in urban areas due to urbanization [17,20,32]. Almost all the studies reported an urban advantage, though a few reported that those living in urban slums were worse off than their rural counterparts [27,28]. Only one multi-country study [23] found that the urban poor had higher U5M than those living in rural areas. Multi-country studies show a distinct advantage in urban areas in controlling U5M compared to rural areas. However, single country or state level studies show convergence between the rural and urban U5M rates, largely due to loss of progress in urban areas, or due to higher progress in rural areas. This means that efforts made in rural areas are not complimented with work done in urban areas. Also, rural disadvantage persisted in most of the middle- and low-income countries because the percentage of the rural population was high.

The meta-analysis determined that the combined effect size for the relative risk of the 15 studies was 1.47. This means that during the MDG period, there was a rural disadvantage, since overall, children under the age of five from rural areas in middle- and low-income countries had a 47% increased risk of dying before the age of five years compared to those in urban areas. This is in keeping with studies done before the MDG period that report an increased risk of U5M living in rural areas [40]. The meta-analysis suggests a positive association between those living in rural areas and U5M, suggesting that children born to mothers in rural areas were at a disadvantage in surviving until their fifth birthday compared to those born in urban areas.

Publication bias may be present, based on the distribution of studies in the funnel plot. Publication bias refers to the fact that studies that fail to find an association between rural place of residence and U5M have less chances of being published than studies that show a positive or statistically significant association. However, asymmetry in a funnel plot is only an indication of publication bias and not conclusive evidence. In addition, meta-analysis free from publication bias may have an asymmetric funnel plot for other reasons [41]. Seeing that heterogeneity was determined to be low in the present study, more likely than not the asymmetry observed in the funnel plot may be due to reporting bias, since we used only published data. Nonetheless, the present study may be instructive in supporting future efforts to reduce U5M in rural areas.

Sensitivity analysis indicated that this rural disadvantage held true even when successive studies were omitted, and the combined RR was greater than 1. This provides evidence as to the robustness of the results.

The meta-regression analysis returned a random effect model where an increase in the percentage of rural population led to an increase in the relative risk of U5M. Though this was not significant, the positive relationship between U5M and the percentage rural population cannot be ignored. Efforts must be continued to remove this disadvantage by making improvements in health facilities in rural areas. This is important if the SDG are to be met. Distance to health care and cost of health care should also be considered in any policy planning [42].

## 5. Limitations

There were a couple of notable limitations to the present review. Rural and urban areas are country-specific and most multi-country analyses do not take into account any differences in country-wise definitions. Also, the review dealt only with rural/urban places of residence. Other variables such as wealth quintile and maternal age, that may also have a significant association with U5M, were not considered.

## Figures and Tables

**Table 1:** Characteristics of included studies.

Characteristics	Number of Studies
**Regions**	
Africa	1
Sub-Saharan Africa	14
South Asia	5
Southeast Asia	2
Middle East	2
Global (low income/developing countries)	1
Southwestern Pacific	1
**Place of Residence**	
Both urban and rural	26
**Source of Data**	
Demographic health survey (DHS)	16
Census	1
Others	2
Combination of Surveys	7

**Table 2:** Selected studies showing regions and measures.

Study	Country/Region	Measure	Source of Data	Study Period (years)	Results	Rural Population (%)	Notes
Khawaja et al. [16]	18 Middle eastern countries	Rural deaths/100 urban	DHS 1990–1998	1990–1999	112–231	29	Urban advantage observed.
Minnery et al. [17]	India	U5M	NFHS (National Family Health Survey) 1992–1993, 1998–1999, 2005–2006 DLHS (District Level Household and Facility Surveys 1998–1999, 2002–2004, 2007–2008 SRS (Sample Registration System 1971–2008	1990–2007	R 81–144 U 50–78	72	Study on two states in India, Chattisgarh and Jharkhand. Notes disparities between urban and rural place of residence and U5M at district level. Notes lower progress in reduction of U5M in urban areas.
Nguyen et al. [18]	Nepal	RR RD	NDHS (Nepal Demographic Health Survey) 1996, 2001, 2006 NLSS I (Nepal Living Standards Survey) 1995–1996, NLSS II 2003–2004	1990–2005	1.27–1.58 13.8–40.4	72	Inequities in rural/urban location. Urban children have a better chance of survival than those born in rural areas.
Wang [7]	60 Low income countries	U5M	DHS 1990–99, WDI (World Development Indicators)	1990–1999	R 34–347 U 34–210	56	Gap in U5M in rural and urban areas. The assumption is that most poor live in rural areas. Rural areas have slower reduction in U5M than urban areas. Overall, in rural areas, U5M has reduced from 143 in 1990 to 126 in 1999. In urban areas, U5M has reduced from 105 in 1990 to 89 in 1999.
Garenne [8]	31 African countries	RR	47 DHS and WFS (World Fertility Survey)	1950–2000	0.94–2.03	72	Reported no change in status in 19 countries; convergence in 11 countries; and divergence in 1 country in the association of U5M and rural and urban areas of residence.
Kayode et al. [19]	Nigeria	OR	Nigeria DHS 2008	2003–2008	R 1.53 U 1.00	51	Living in urban areas reduced the odds of U5M.
Nguyen et al. [20]	India	U5M	NFHS 1992–1993, 1998–1999, 2005–2006, DLHS 1998–1999, 2002–2004, 2007–2008, SRS 1971–2008, WHS (World Health Survey) 2003	1990–2007	MadhyaPradesh R 104–178 U 67–97 Orissa R 92–150 U 68–98	88	Data on two states, Madhya Pradesh and Orissa. Convergence between rural and Urban U5M rates; attribute to largely inadequate progress in urban areas.
Kazembe et al. [21]	Rwanda, Senegal and Uganda	HR BV(MV)	Census data 2001 (Rwanda) 2002 (Senegal, Uganda)	2001–2002	Rwanda U 0.67 (MV 0.79) Senegal 0.63 (MV 1.01) Uganda 0.68 (MV 1.04)	77	Three sub-Saharan countries. Lower deaths associated with living in urban areas.
Anyamele. [22]	11 Sub-Saharan African countries	RR	DHS 2003–2007	2000–2006	1.16–1.66	71	Those born in Urban areas have better odds of survival past their fifth birthday than those in rural areas. Calculated the ratio of rural to urban areas and U5M. Report a wide difference.
Van de Poel et al. [23]	47 Developing Countries	RR	DHS 1994–2004	1989–2003	0.72–1.80	62	Considerable rural–urban gap in child health outcomes. Higher U5M in rural areas. In a number of countries urban poor has higher mortality than rural counterparts. Controlling for wealth, the rural urban gap remains significant in 17 countries.
Tran et al. [24]	Papua New Guinea	U5M	DHS 1996, 2006 National Census 2000	1985–1999	R 83–92 U 32–38	86	Lower U5M in urban areas. National estimates closer to rural estimates. Estimates at provincial and district level. Reduction in U5M lower in rural areas. Poverty a significant indicator.
Sreeramareddy et al. [25]	Nepal	U5M	DHS 1996, 2001, 2006, 2011	1991–2010	R decreased from 123 to 56. U decreased from 73 to 44.	86	Relative and absolute inequalities for U5M reduced for rural/urban. Decrease higher in rural areas.
Jimenez-Soto et al. [26]	Cambodia	Rr and RD Base = Urban	DHS 2000, 2005, 2010	1995–2010	Rr increased from 1.56 in 1989–90 to 2.41 in 2009–2010. RD 31–46	79	U5M decreasing however inequalities increasing. Rural disadvantage observed.
Hodge et al. [27]	Indonesia	Rr and RD Base = Urban	7 IDHS 1980–2011 (1987, 1991, 1994, 1997, 2002–03, 2007–2008, 2012	1980–2011	Rr 1.24–1.75 RD 15.5–40.3	46	Decline in national U5M and decline in absolute inequality in rural/urban location. Rural population migrating into urban slums may have caused closing gap.
Kimani-Murage et al. [28]	Kenya	U5M	KDHS (Kenya DHS) 1993, 1998, 2003, 2008, NUHDSS (Nairobi Urban Health and Demographic Surveillance System) 2003–2010	1979–2008	R 73–105 U 69–100	82	Disparities narrowing because of more rapid decline in U5M in rural than urban areas. But they report higher U5M in urban slums than in rural and non-slum urban areas.
Daniel [29]	Ghana	U5M	Ghana DHS 2008	2003–2007	R 90 U 75	51	U5M rates were consistently higher in rural areas. 62.5% greater chance of dying in rural areas compared to urban areas.
Sayem et al. [30]	Bangladesh	U5M	Bangladesh DHS 2007	2007	U 63 R 77	72	U5M decline is faster in urban than in rural areas. Rural poor are more vulnerable population that needs more attention.
Negera et al. [31]	Ethiopia	U5M	DHS 2000, 2005, 2011	1995–2011	U 149 to 83 R 193 to 114	84	U5M higher in rural areas for the 3 survey periods under study.
Amouzou [32]	Sub-Saharan Africa	U5M	DHS	1960–2000	% Urban Bivariate model −0.02 Main effects model −0.01 Extended model −0.01	71	Negative association between urbanization and U5MR observed.
Van Malderen et al. [33]	Africa	Percentage Under-five deaths	DHS 2007–2010	2002–2007	U 2.7–11.9 R 2.4–12.5	60	13 African countries. U5M higher in rural areas. Focus on wealth related inequalities.
Ettarh and Kimani. [34]	Kenya	Multivariate HR	DHS 2008–2009	2003–2009	3.61 R compared with U R 1040/13149 U 167/3013	82	Likelihood of death in rural areas significantly higher than in the urban areas.
Kaldewei [35]	Jordan	U5M	JPFHS (Jordan Population and Family Health Survey) 2007	2002–2006	R 27 U 22	18	Urban advantage persists even though the rural population is only 16.5% in the survey. Overall, MDG4 targets to be achieved.
Ndawala [36]	Malawi	U5M	DHS 2000	1991–2000	10-year estimates U 147.9 per 1000 R 210.4 per 1000	85	Rural mortality rates higher than urban. The rural-urban differential larger in the neonatal period than the postneonatal period.
Adedini et al. [37]	Nigeria	HR	DHS 2008	2003–2008	Rural = Model 5 1.22 (1.07 1.38), Model 6 1.21 (1.07 1.37) Model 7 1.23 (1.08 1.39) Model 8 1.21 (1.08 1.37) For all urban =1	51	All models were significant and indicated a rural disadvantage for children under-five.
Dejene and Girma [38]	Ethiopia	HR	DHS 2011	2001–2010	Unadjusted U 1 R 1.4 (1.24, 1.58) Adjusted U 1 R 1.02 (0.86, 1.21)	80	Urban advantage however gap narrowing.
Corker [39]	12 Sub-Saharan African countries	Kaplan-Meier Survival Estimates	DHS 1995–2000, 2005–2010	1990–2010	Change in AD-0.006% U-R R 1.045 in 1995–2000 To R 1.037 in 2005–2010	64	12 Sub-Saharan countries, the urban advantage remains but decreases slightly. Urbanisation poses new threat. Higher gains in rural areas led to decrease in inequalities in U5M.

R: rural, U: urban, RD: rate difference, BV: bivariate, MV: multivariate, RR: relative risk, OR: odd ratio, HR: hazard ratio, Rr: rate ratio, U5M: under-five mortality, NFHS: National Family Health Survey, DLHS: District Level Household & Facility Survey, DHS: Demographic Health Survey, all studies are referred to by the first author’s name only.

**Table 3:** Results from the meta-regression (random effects model).

Variable	*β*	95% CI for *β*		Standard Error	*p-value*
		**Lower**	**Upper**		
Constant	0.988	0.038	1.94	0.485	0.041
Rural population (%)	0.007	−0.006	0.02	0.007	0.3

CI: confidence interval.

**Table 4:** Results from the sensitivity analysis showing the effect size and the respective confidence intervals.

Study Omitted	Effect Size (RR)	CI
Kimani-Murage et al. [28]	1.50	1.29–1.71
Daniel [29]	1.49	1.29–1.70
Van de Poel et al. [23]	1.48	1.26–1.71
Garenne [8]	1.48	1.26–1.70
Wang [7]	1.48	1.26–1.69
Anyamele [22]	1.48	1.26–1.69
Sreeramareddy et al. [25]	1.47	1.26–1.70
Nguyen et al. [18]	1.47	1.25–1.68
Minnery et al. [17]	1.46	1.24–1.67
Tran et al. [24]	1.41	1.33–1.49
Nguyen et al. [20]	1.48	1.26–1.69
Sayem et al. [30]	1.49	1.28–1.70
Negera et al. [31]	1.48	1.27–1.70
Kaldewei [35]	1.49	1.28–1.69
Ndawala [36]	1.48	1.27–1.69

CI: confidence interval, RR: relative risk.
